# Management of unstable pertrochanteric fractures, evaluation of forgotten treatment options

**DOI:** 10.1051/sicotj/2020020

**Published:** 2020-06-24

**Authors:** Amr Ahmed Hosny Abdo Selim, Fady Kamal Beder, Ibrahim Taha Algeaidy, Ahmed Samir Farhat, Nader M. Diab, Ahmed Samir Barakat

**Affiliations:** 1 Department of Orthopaedic and Trauma Surgery, Faculty of Medicine, Cairo University Cairo Egypt

**Keywords:** Unstable pertrochanteric fracture, Hip fractures, Elderly, Dynamic Hip Screw DHS, Trochanteric Stabilization Plate TSP, Proximal femoral locked plate PFLP

## Abstract

*Introduction*: Unstable trochanteric fractures are challenging with a high rate of implant failure and re-operation. Cephalomedullary nails proved to be a rational management choice for these injuries, yet other management options have not been well assessed. The aim of this study was to compare the use of DHS with trochanteric stabilizing plate (TSP) and proximal femoral locked plate (PFLP) in unstable pertrochanteric fractures. *Methods*: This randomized controlled trial (RCT) included 40 patients (22 males, 18 females) with unstable pertrochanteric fractures (AO/OTA 31A2.2/A2.3). The patients were randomized into group 1 managed by DHS with TSP while group 2 was managed by PFLP. All patients were followed up for 1 year. Patients were assessed radiographically and clinically using Harris hip score (HHS) at 3, 6 and 12 months. Operative time, estimated blood loss and time to union were also compared. *Results*: The difference of intra-operative variables, including operative time and intra-operative blood loss, between both groups was statistically insignificant. Time to bony union was faster in the first group with a statistically significant *P* value (*p* = 0.04). Functional outcome per HHS was significantly better in group 1 (*p* < 0.01) and implant failure in group 1 occurred statistically lesser (*p* < 0.01). *Discussion*: DHS with TSP appears to be a good option of management for unstable pertrochanteric fractures. In contrast, the use of PFLP in unstable pertrochanteric fractures in the elderly does not appear to be a good alternative.

## Introduction

Pertrochanteric femoral fractures are common injuries affecting the elderly population. They account for more than 50% of all hip fractures and are a common orthopaedic problem encountered in this age group [1], and are associated with considerable morbidity and mortality [2, 3].

There are many classification systems for these fractures. Prototypical is the Evans classification which divides them into stable and unstable fractures based on the fracture pattern [4]. Accordingly, fractures are defined as stable if they are two parts and unstable if they three or four parts. The AO/OTA classifies trochanteric fractures into three groups: 31 A1 simple pertrochanteric, A2 multi-fragmentary pertrochanteric and A3 intertrochanteric patterns. A2 type is further sub-divided into A 2.2 with one intermediate fragment and A2.3 with two or more intermediate fragments; both of them are considered unstable pertrochanteric fracture patterns [5].

Forty percent of pertrochanteric femoral fractures are unstable and bear a higher failure rate when managed by conventional treatment options, such as DHS and cephalomedullary nails when compared to stable patterns. This instability is multi factorial and includes loss of the posteromedial calcar support, loss of the posterolateral support or lateral femoral wall insufficiency [6].

Thus, lateral wall reconstruction plays an imperative role in maintaining stability of these fractures and hence on the functional outcome. By providing a buttress effect to the lateral wall of the proximal fragment, excessive fracture collapse, significant limb shortening, varus malposition, and medialization with eventual fixation failure are prevented [7].

DHS has long been the preferred modality of stable trochanteric femoral fracture management; nevertheless, proximal nail devices have replaced the former in many parts of the world. Yet, there is a high DHS failure rate with unstable trochanteric femoral fractures. Consequently, various fixation devices have been introduced to replace DHS in unstable fracture patterns. These include cephalomedullary nails, the add-on trochanteric stabilizing plates, fixed angle blade plates, and proximal femoral locked plates [8, 9]. Cephalomedullary nails have proven to be a reasonable option of management for unstable pertrochanteric fractures; however, few studies have evaluated the use of DHS with TSP and PFLP as alternative management options.

Trochanteric stabilizing plates (TSP) are used to stabilize the greater trochanter and lateral wall and they are thought of a modular extension of the DHS. Fixation of unstable intertrochanteric fractures by the TSP augmented DHS have been noted to have lesser incidence of femoral medialization and greater improvement in the functional outcome [10].

On the other side, the proximal femoral locked plate (PFLP) provides a good lateral wall buttress. Moreover, its locking capability along with its possible minimal-invasive insertion technique brands it as an apparently attractive alternative to other fixation devices [11].

This works compares the functional and radiological outcome of a series of patients with unstable pertrochanteric fractures treated with either DHS + TSP or PFLP. Surprisingly, no comparison between these two management options was found in the literature.

## Materials and methods

This study was conducted in the period from January 2016 to December 2018 after having obtained Ethical Board approval.

It involved 40 patients aged from 60 to 90 years. Inclusion criteria were patients with unstable pertrochanteric fractures AO 31A2.2 / AO 31A2.3. Excluded were patients with open fractures and with pre-operative neurological deficits. Full clinical and radiological examinations were performed to all patients in the Accident & Emergency (A&E) department.

The needed study sample for a power of 90% and permissible error of 5% is 40.

Informed consent was obtained from all patients. Patients were randomized using a software-based randomizer (https://www.randomizer.org) into two groups. Group 1 included patients managed by DHS with TSP, while group 2 included patients managed by PFLP.

Patients were planned to be operated on the same day of admission, otherwise they were postponed based on other medical causes like elevated blood pressure or uncontrolled blood glucose levels which occurred in nine cases. Overall there were nine patients with hypertension and eight with Type II diabetes mellitus in group 1, and two patients with hypertension and seven with diabetes in group 2. All the surgeries were done by the three senior authors.

**Figure 1:**
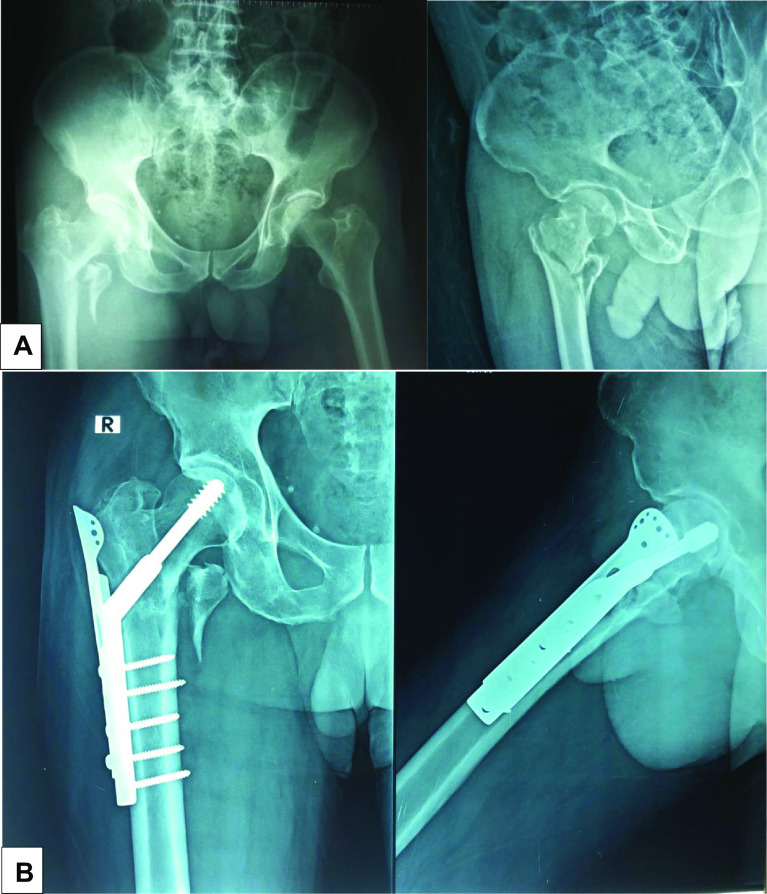
X-rays of 63-year-old male patient with AO 31A2.2 pertrochanteric fracture (lateral wall thickness < 20 mm) managed by DHS with TSP: (A) Pre-operative X-rays; (B) X-rays at 6 months follow-up.

In both groups a fracture table was utilized with the patient in the supine position. Closed reduction was performed in all cases by gentle traction with internal rotation, and a lateral approach to the proximal femur was utilized. In group 1, the guide wire of the DHS was applied aiming to be central in AP and lateral views. The triple reamer was then used followed by taping the track before applying the DHS lag screw. A tip-apex-distance of no more than 25 mm was intended and realized in all cases. A four or five-hole DHS plate was then inserted and stabilized to the shaft by applying a screw through the second hole. The trochanteric stabilizing plate was placed over the DHS plate and fitted by putting its large oval hole opposite to the DHS lag screw. Next it was secured by applying a screw into the 3rd hole distally. According to the bone quality, the proximal part of the TSP was fixed to the greater trochanter by either Ethibond 2 (5.0 metric) sutures or 4-mm Cancellous screws. The distal screw was then inserted from the TSP into the femoral shaft (Figure 1). Closure was done in layers starting by continuous watertight sutures for the ilio-tibial band.

**Figure 2:**
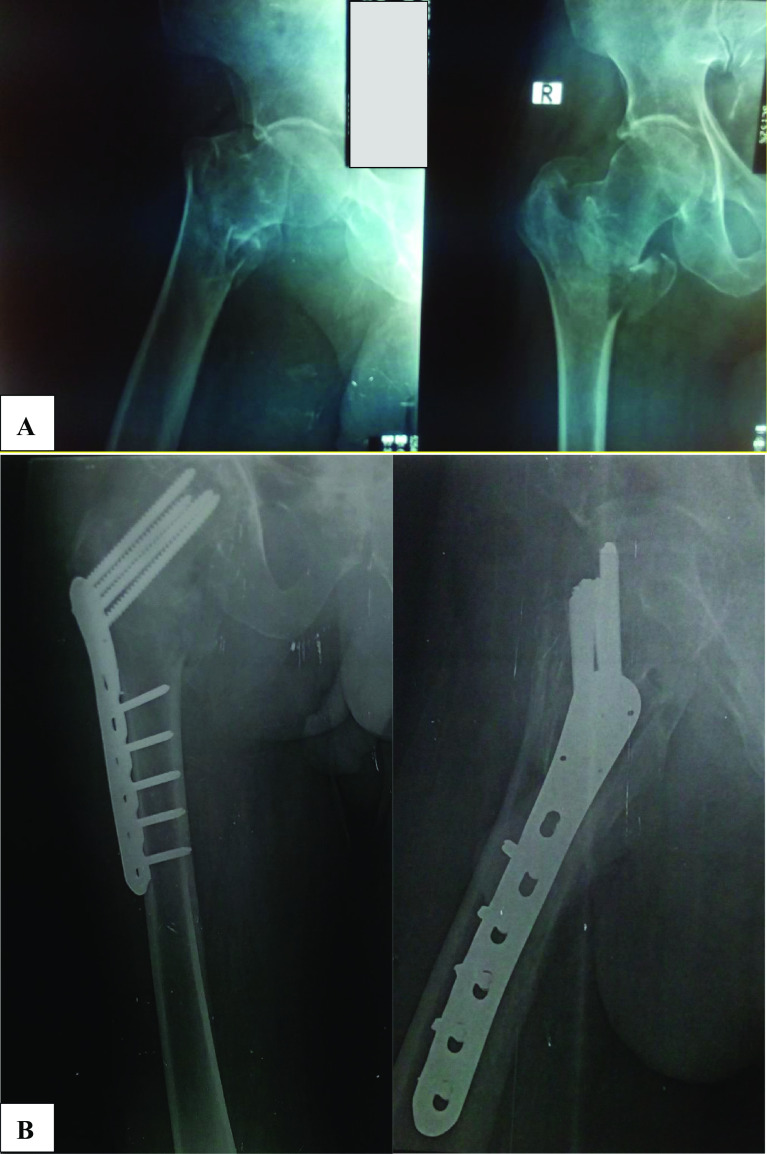
X-rays of 62-year-old male patient with AO 31A2.2 pertrochanteric fracture managed by PFLP; (A) Pre-operative X-rays; (B) X-rays at 6 months follow-up.

In group 2 positioning, reduction and the surgical approach were done analogously. The Proximal Femoral Locking Plate was placed and fitted against the lateral aspect of the femoral shaft. Correct plate positioning was obtained by applying K-wires through the proximal holes and checking their position in AP and lateral views. Once the plate was correctly positioned to the proximal part, one screw was inserted from the distal holes into the shaft without overtightening it just to secure the plate in position. The proximal K-wires were then removed systematically and replaced by cancellous locking screws. Finally, multiple locking screws were inserted from the distal holes into the femoral shaft (Figure 2). Closure was done as in the first group.

Immediate weight bearing as tolerated was allowed for all patients under physiotherapeutic guidance utilizing a Zimmer frame.

Patients were followed-up clinically and radiologically for a minimum of 1 year at regular intervals of 2 weeks, 6 weeks, 3 months, 6 months and at 1 year when they were subjected to the final clinical and radiological evaluation.

Clinical outcome was assessed using the Harris Hip Score (HHS) at 6 weeks, 3 months, 6 months and 1 year. Radiological outcome was assessed based on progression of union in hip AP and lateral views. Union was defined radiologically by progression of callus or disappearance of the fracture lines in three out of four cortices, and clinically by disappearance of pain at the fracture site by palpation and on weight bearing. Operative time defined as skin to skin and estimated intraoperative blood loss were recorded.

SPSS 23.0 (IBM, Armonk, NY, USA) was used for statistical analysis. Quantitative variables were expressed in the form of mean, standard deviation (SD), minimum and maximum and qualitative variables in the form of numbers (No.) and percentages (%). Data were explored for normality using Kolmogorov–Smirnov test of normality, which showed that the data were normally distributed. The confidence interval was set to 95% and the *P* value to 0.05. Pearson correlation coefficient (*r*) was used to analyse the degree of association between two variables. Multivariate ANOVA was done followed by univariate ANOVA using Wilks’ *λ* equation. Bonferroni correction to compensate for multiple ANOVAs was used and the statistical significance level was set for MANOVA calculations to *p* < .025.

## Results

The study sample included 22 males (55%) and 18 females (45%) with a mean age of 69.03 years. The mode of trauma was fall to the ground in 30 (75%), and road traffic accident in 10 of the patients (25%). The right side was affected in 22 (55%) patients and the left in 18 patients (45%). Two patients (5%) had associated fractures in the form of non-displaced distal radius fractures which were treated by cast application and were distributed evenly in each group.

The mean intra-operative time was 116.75 ± 32.49 min in group 1, while group 2 had a mean intra-operative time of 118.25 ± 16.72 min (*p* = 0.85). Intra-operative blood loss in group 1 averaged 312.50 ± 87.54 mL, while group 2 had a mean blood loss of 325.00 ± 93.71 mL (*p* = 0.66). Hospital length of stay in group 1 averaged 8.15 ± 2.66 days, while group 2 had a mean hospital stay of 7.90 ± 1.77 days (*p* = 0.72). Comparing these data showed no difference in the operative and peri-operative variables between both groups with statistically insignificant *P* values.

Time to bony union differed significantly and averaged 14.47 ± 5.37 weeks in group 1, and 17.67 ± 3.37 weeks in group 2 (*p* = 0.04).

**Figure 3:**
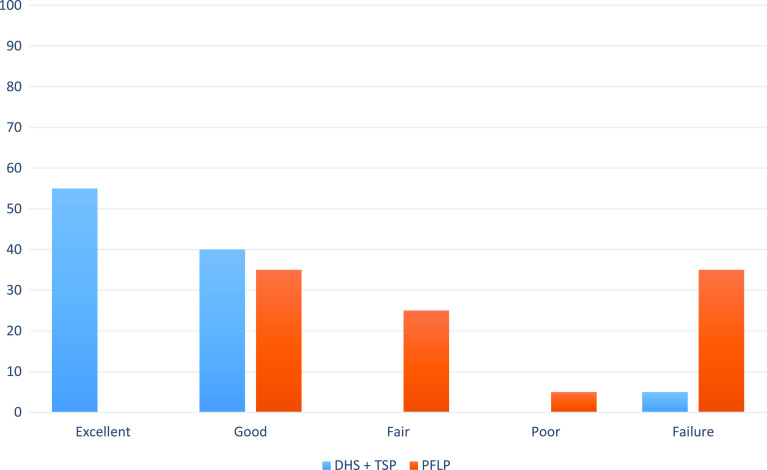
Comparison of Harris hip scores and Failure rate at 1 year between the two groups (in percentages).

Group 1 had a mean HHS 72.53 ± 12.42 at 3 months, meanwhile group 2 had a mean HHS 62.50 ± 8.211. The mean HHS at 6 months for the first group was 83.32 ± 9.73; however, it was 76.38 ± 7.289 in group 2. The mean HHS at 1 year for the first group was 89.42 ± 6.04, and 78.38 ± 7.482 in group 2 (Figure 3). Comparing the HHS of both groups showed statistically significant *P* values at 3 months, 6 months and 1 year of <0.01 (Table 1).

**Table 1:** Functional outcome per Harris Hip Score at 3, 6 and 12 months.

		Mean	SD	Median	Minimum	Maximum	*P* value
Harris Hip Score (3 months)	DHS + TSP	72.53	12.42	71	49	100	< 0.01
	PFLP	62.50	8.211	70	50	74	
Harris Hip Score (6 months)	DHS + TSP	83.32	9.73	84	70	100	< 0.01
	PFLP	76.38	7.289	80	66	85	
Harris Hip Score (12 months)	DHS + TSP	89.42	6.04	90	80	100	< 0.01
	PFLP	78.38	7.482	85	68	89	

DHS: Dynamic Hip Screw; TSP: Trochanteric Stabilizing Plate; PFLP: Proximal Femur Locked Plate.

Prior to MANOVA testing the dependent variables were tested for normal distribution. A one-way MANOVA revealed a significant multivariate main effect for implant type, Wilks’ *λ* = 0.405, *F*(3, 23) = 11.256, *p* < 0. 000, and partial *λ* ^2^ = 0.595. Power to detect the effect was 0.997. Due to the significance of the overall test, univariate main effects were examined. Significant univariate main effects for HHS were obtained for utilized implant type. *F*(1, 25) = 4.358, *p* = 0.047, partial *λ* ^2^ = 0.148, and observed power = 0.519 at 3 months HHS and *F*(1, 25) = 3.266, *p* = 0.083, partial *λ* ^2^ = .116 and observed power = .412 at 6 months and HHS at 12 months *F*(1, 25) = 16.378, *p* < .0001, partial *λ* ^2^ =.396 and observed power .973. Gender, preexisting morbidities, concomitant fractures and site affection had no effect on the dependent outcome variables.

Regarding complications, superficial infection occurred in 1 case (5%) in each group. Deep infection ensued in 1 case (5%) in group 1, while in 2 cases (10%) in group 2. Ultrasonographic confirmed DVT affected 1 case (5%) in group 2. These findings were statistically insignificant.

Finally implant failure rate with subsequent non-union, mechanical deformity and need for revision occurred in 1 (5%) patient in group 1 and in 7 patients (35%) in group 2 which was statistically significant (*p* = 0.01). In group 1 implant failure was in the form of lag screw cutout, whereas in group 2 it was in the form of screw back out, screw breakage, plate bending and plate breakage (Table 2). The presence of hypertension and diabetes mellitus had no statistical verifiable influence on the rate of complications.

**Table 2:** Implant failure rate and types in both groups.

			Patients and (%)		*P* value
Implant complications	DHS + TSP	Lag screw cut-out	1 (5%)		0.01
	PFLP	Bending of the plate	4 (20.0%)	7 (35%)	
		Screw back out	1 (5.0%)		
		Screw breakage	1 (5.0%)		
		Plate breakage	1 (5.0%)		

DHS: Dynamic Hip Screw; TSP: Trochanteric Stabilizing Plate; PFLP: Proximal Femur Locked Plate.

## Discussion

The present study revealed that DHS with TSP had a superior functional outcome expressed by HHS, smaller implant failure rate, and required a shorter time to union when compared to PFLP. However, there was no significant difference regarding the operative time, blood loss, hospital stay or infection rate.

No statistical difference regarding the rate of reoperation was reported in a meta-analysis conducted by Arirachakaran et al. which encompassed 36 studies to compare the outcomes of PFNA, Medoff plate, less invasive stabilization system (LISS), percutaneous compression plating (PCCP), Gamma nails, PFNA and DHS. Due to the shortened operative time, decreased need for blood transfusion and decreased rate of general and wound complications, they concluded that PCCP was the best choice for intertrochanteric fractures [12]. Nevertheless, a proper weighting for unstable fractures in this meta-analysis was not made and therefore their finding might be misleading as the treating surgeon has to undoubtedly differentiate between the simple stable and the more complex unstable fracture pattern.

Shetty et al. conducted a study of 32 patients with unstable trochanteric fractures, all of them underwent DHS with TSP fixation. They assessed the functional outcome using HHS and the radiological outcome by means of the RUSH score. 15 patients (46%) had poor bony union, while 17 (53.5%) patients had good bony union. Regarding the functional outcome; 9 (28%) patients had excellent results, 10 (31%) had good, nine (28%) had fair and four (12.5%) had poor outcome [13]. However, this study lacked the comparative quality with other management options (Table 3).

**Table 3:** Comparison between our study and other relevant studies.

	DHS with TSP						PFLP					
	Bony union	HHS (Excellent, good, fair and poor, from left to right) by percentages at final follow-up of the study.				Failure	Bony union	HHS (Excellent, good, fair and poor, from left to right) by percentages at final follow-up of the study.				Failure
Shetty et al.	53.5% at 6 months	28	31	28	12	Poor union in 46%						
Asif et al.							92% at 1 year.	88		12		8%
Shah et al.							18.75 W	50	15	15	20	45%
Raman et al.	100% at 15.23 W	65.5	34.5	0	0	No						
Our study	14.47 W	55	40	0	0	5%	17.67 W	0	35	25	5	35%

DHS: Dynamic Hip Screw; TSP: Trochanteric Stabilizing Plate; PFLP: Proximal Femur Locked Plate; HHS: Harris Hip Score; W: weeks.

Asif et al. conducted a study encompassing 62 patients with unstable trochanteric fractures managed by PFLP or DHS. Of the 27 patients treated with PFLP, 25 patients were evaluated for final outcome; 23 (92%) of them showed union at 1-year follow-up. One patient (2%) developed bending of proximal screws and three (6%) developed varus collapse. From the 35 patients treated with DHS, eight patients (22%) developed varus collapse, seven (20%) developed medialization and three (8%) had femoral head screw cut out. Regarding the HHS in the PFLP group 88% had good to excellent results whereas 60% cases had good to excellent results in the DHS group [14]. The limiting factor of this study, however, was that it compared the PFLP to the DHS as stand-alone implant.

Shah et al. reported of 20 cases with unstable trochanteric fractures treated with PFLP. The average time of union was 18.75 ± 3.67 weeks. Ten (50%) cases had excellent Harris Hip Scores, 3 (15%) cases had good, 3 (15%) cases had fair and 4 (20%) cases had poor functional scores with an average HHS of 80.2 ± 28.54. The complication rate was 45% which included four cases (20%) of superficial infection, two cases (10%) of deep infection and four cases (20%) of late complications including non-union as well as mechanical deformation [15].

In an interesting approach to provide more lateral wall stability Gadegone et al. applied either an additional screw from the greater trochanter to the inferior sector of the femoral head or a cerclage wire around the proximal femur to strengthen the lateral trochanteric wall over a proximal femoral nail. In their prospective series of 82 patients with an average follow-up of 8.4 months they reported a mean healing time of 14.2 weeks. Five patients developed lateral migration of the neck screws (6.1%), infection in 2 patients (2.44%), Z-effect in one (1.22%) and fracture of the distal interlocking bolt in another patient (1.22%). They concluded that screw or cerclage augmentation of a standard PFN increases stability [16].

Raman et al. reported 58 patients with unstable intertrochanteric fractures who were treated using DHS and TSP. Bony union was achieved in all the cases at an average of 15.23 weeks. Thirty-eight patients (65.5%) had excellent scores above 90 at the final follow-up and the rest (34.5%) had HHS above 80 [17].

In this study the TSP augmented DHS group showed a 5% implant failure rate with subsequent non-union, and this is significantly less than the study of Shetty et al. which reported a complication rate of 46% related to osseous union. The bony union was faster in our study (14.47 weeks) in comparison to the study done by Shetty et al. (24 weeks), and the functional outcome using HHS was better in our study. However, our study has a higher implant failure rate than the study conducted by Raman et al.

Regarding the PFLP, our study showed a smaller implant failure rate (35%) than the study conducted by Shah et al. (45%), yet a higher implant failure rate than the study conducted by Asif et al. (8%).

The concept of lateral wall thickness in pertrochanteric fractures as an outcome predictor is currently discussed extensively. A minimum of 20.5 mm–22 mm has been recommended to avoid lateral wall failure and subsequent loss of reduction when using a DHS device [18, 19]. To the best of our knowledge the applicability of this concept to intramedullary devices has yet not been investigated.

Yet, the limited patient number, short follow-up and the single center nature of the underlying study are clear confounding factors. Moreover, the degree of pre-existing osteoporosis and the biomechanical analysis were not considered.

## Conclusion

DHS with TSP is a valid option of management for unstable pertrochanteric fractures and should always be put into consideration alongside cephalomedullary nails. In contrast, the use of PFLP in unstable pertrochanteric fractures in the elderly does not appear to be a good option.

## Conflict of interest

The authors declare that there are no potential conflicts of interest or financial relationships relevant to this research.

## References

[1] Dhanwal DK, Dennison EM, Harvey NC, Cooper C (2011) Epidemiology of hip fracture: Worldwide geographic variation. Indian J Orthop 45(1), 15–22. 21221218 10.4103/0019-5413.73656 PMC3004072

[2] Lavini F, Renzi-Brivio L, Aulisa R, Cherubino F, Di Seglio PL, Galante N, Leonardi W, Manca M (2008) The treatment of stable and unstable proximal femoral fractures with a new trochanteric nail: results of a multicentre study with the Veronail. Strat Traum Limb Recon 3(1), 15–22. 10.1007/s11751-008-0035-y PMC2291480 18427919

[3] Tawari AA, Kempegowda H, Suk M, Horwitz DS (2015) What makes an intertrochanteric fracture unstable in 2015? Does the lateral wall play a role in the decision matrix? J Orthop Trauma 29(Suppl 4), S4–9. 25756825 10.1097/BOT.0000000000000284

[4] Andersen E, Jørgensen LG, Hededam LT (1990) Evans’ classification of trochanteric fractures: an assessment of the interobserver and intraobserver reliability. Injury 21(6), 377–378. 2276801 10.1016/0020-1383(90)90123-c

[5] Buckley RE, Moran CG, Apivatthakakul T (2018). AO Principles of Fracture Management (Third). Stuttgart: Georg Thieme Verlag 2, p. 774.

[6] Voleti PB, Liu SY, Baldwin KD, Mehta S, Donegan DJ (2015) Intertrochanteric Femur Fracture Stability. Geriatric Orthop Surg Rehab 6(3), 192–196. 10.1177/2151458515585321 PMC4536511 26328235

[7] Gotfried Y (2004) The lateral trochanteric wall: a key element in the reconstruction of unstable pertrochanteric hip fractures. Clin Orthop Relat Res 425, 82–86. 15292791

[8] Gupta RK, Sangwan K, Kamboj P, Punia SS, Walecha P (2010) Unstable trochanteric fractures: the role of lateral wall reconstruction. Int Orthop 34(1), 125–129. 19288102 10.1007/s00264-009-0744-y PMC2899273

[9] Bajpai J, Maheshwari R, Bajpai A, Saini S (2015) Treatment options for unstable trochanteric fractures: Screw or helical proxima femoral nail. Chin J Traumatol = Zhonghua Chuang Shang Za Zhi 18(6), 342–346. 26917025 10.1016/j.cjtee.2015.03.006

[10] Hsu C-E, Chiu Y-C, Tsai S-H, Lin T-C, Lee M-H, Huang K-C (2015) Trochanter stabilising plate improves treatment outcomes in AO/OTA 31–A2 intertrochanteric fractures with critical thin femoral lateral walls. Injury 46(6), 1047–1053. 25890863 10.1016/j.injury.2015.03.007

[11] Kumar N, Kataria H, Yadav C, Gadagoli BS, Raj R (2014) Evaluation of proximal femoral locking plate in unstable extracapsular proximal femoral fractures: Surgical technique & midterm follow up results. J Clin Orthop Trauma 5(3), 137–145. 25983487 10.1016/j.jcot.2014.07.009 PMC4223809

[12] Arirachakaran A, Amphansap T, Thanindratarn P, Piyapittayanun P, Srisawat P, Kongtharvonskul J (2017) Comparative outcome of PFNA, Gamma nails, PCCP, Medoff plate, LISS and dynamic hip screws for fixation in elderly trochanteric fractures: a systematic review and network meta-analysis of randomized controlled trials. Eur J Orthop Surg Trauma 27(7), 937–952. 10.1007/s00590-017-1964-2 28434124

[13] Shetty A, Ballal A, Sadasivan AK, Hegde A (2016) Dynamic hip screw with trochanteric stablization plate fixation of unstable inter-trochanteric fractures: a prospective study of functional and radiological outcomes. JCDR 10(9), RC06–RC08. 10.7860/JCDR/2016/20275.8415 PMC5072031 27790531

[14] Asif N, Ahmad S, Qureshi OA, Jilani LZ, Hamesh T, Jameel T (2016) Unstable intertrochanteric fracture fixation – Is proximal femoral locked compression plate better than dynamic, hip screw? JCDR 10(1), RC09-13–13. 10.7860/JCDR/2016/11179.7084 PMC4740662 26894134

[15] Shah MD, Kapoor CS, Soni RJ, Patwa JJ, Golwala PP (2017) Evaluation of outcome of proximal femur locking compression plate (PFLCP) in unstable proximal femur fractures. J Clin Orthop Trauma 8(4), 308–312. 29062210 10.1016/j.jcot.2016.11.005 PMC5647620

[16] Gadegone WM, Shivashankar B, Lokhande V, Salphale Y (2017) Augmentation of proximal femoral nail in unstable trochanteric fractures. SICOT-J 3, 12. 28186871 10.1051/sicotj/2016052 PMC5302881

[17] Raman DDT, Vignesh DA, Swaminathan DS (2018) Clinico-radiological results of unstable trochanteric fractures treated with custom-made trochanteric stabilisation plate and dynamic hip screw (DHS). Intl J Orthop Sci 4(3.3), 308–313.

[18] Kumar R (2015) The role of lateral femoral wall thickness in intertrochanteric fracture. J Evid Based Med Healthcare 2, 842–844.

[19] Hsu CE, Shih CM, Wang CC, Huang KC (2013) Lateral femoral wall thickness: A reliable predictor of post-operative lateral wall fracture in intertrochanteric fractures. Bone Jt J 95 B, 1134–1138. 10.1302/0301-620X.95B8.31495 23908432

